# Molecular Profile of Locally Aggressive Well Differentiated Thyroid Cancers

**DOI:** 10.1038/s41598-020-64635-8

**Published:** 2020-05-15

**Authors:** Leila J. Mady, Michael C. Grimes, Nayel I. Khan, R. Harsha Rao, Simion I. Chiosea, Linwah Yip, Robert L. Ferris, Yuri E. Nikiforov, Sally E. Carty, Umamaheswar Duvvuri

**Affiliations:** 1 Department of Otolaryngology-Head and Neck Surgery, University of Pittsburgh, Pittsburgh, Pennsylvania US; 2 Division of Endocrinology and Metabolism, Department of Medicine, University of Pittsburgh, Pittsburgh, Pennsylvania US; 3 VA Pittsburgh Health System, Pittsburgh, Pennsylvania US; 4 Division of Molecular and Genomic Pathology, Department of Pathology, University of Pittsburgh, Pittsburgh, Pennsylvania US; 5 Division of Endocrine Surgery, Department of Surgery, University of Pittsburgh, Pittsburgh, Pennsylvania US

**Keywords:** Genetics, Biomarkers, Translational research

## Abstract

Knowledge of the genetic landscape of aggressive well differentiated thyroid cancers (WDTC) is lacking. Retrospective review of institutional database was performed to identify locally-invasive thyroid carcinomas and a comparison cohort of low-risk WDTC. ThyroSeq v2 next-generation sequencing was performed on available tissue. Survival time was analyzed by Kaplan-Meier methods and compared between groups via the log-rank test. Time to recurrence, treating death as a competing risk, was analyzed by cumulative incidence and compared between groups. Of 80 T4 tumors, 29 (36%) were met inclusion criteria, of which, 25 had genetic and clinicopathologic data. Most (24/25, 96%) harbored at least one genetic alteration, most commonly *BRAF* V600E (19, 76%), followed by mutations in the promoter region of *TERT* (14, 56%). Co-occurrence of *BRAF* and *TERT* was identified in 12 (48%) and associated with significantly higher risk of recurrence (p < 0.05). Conversely, co-occurrence of *BRAF* and *TERT* was present in only 5 of 102 (5%) patients presenting with early-stage WDTC. Compared to early-stage WDTC, co-occurrence of *BRAF* and *TERT* mutations are common in locally advanced (T4) thyroid cancer and are associated with an increased risk of recurrence. This knowledge may help predict aggressive behavior pretreatment and inform perioperative decision-making.

## Introduction

Thyroid cancer is the most common endocrine carcinoma, affecting approximately 65,000 patients in the U.S. every year, with the incidence increasing each year^1^. As the use of high-resolution imaging increases, the detection of “incidental” nodules has increased as well, facilitating this “epidemic”. With up to 25–30% of these nodules being categorized as indeterminate pathology as per the Bethesda classification system, the use of genetic analyses has been shown to provide insight into the malignant potential^2^. Studies have elucidated molecular signatures associated with malignant thyroid disease. For example, activation of the MAPK and PI3K-AKT signaling pathways is associated with cancer initiation and progression. *BRAF* point mutations have been correlated with high iodine intake, while *RET/PTC* and *BRAF/AKAP9* chromosomal rearrangements are associated with radiation exposure^3^.

Despite the apparent increase in thyroid cancer, well differentiated thyroid cancer (WDTC) still represents the vast majority of this disease (85–90%). While most well differentiated thyroid carcinomas (WDTC) are indolent tumors associated with low mortality rates, a subgroup of patients experience recurrence which may be associated with more aggressive and even fatal disease^4,5^. Among these high-risk cancers are those associated with locally advanced disease and/or distant metastasis. Although the molecular profile of well differentiated mostly low-risk thyroid cancer is well characterized^6^, the genetic landscape of well differentiated locally advanced thyroid cancers has not been elucidated. This knowledge may help predict such aggressive cancer behavior preoperatively, which may also inform the choice of an appropriate surgical approach and post-surgical management. In this study, we analyzed a consecutive series of T4 thyroid cancers to identify a subset of cancers that were well differentiated and lacked any poorly differentiated or anaplastic carcinoma components. These tumors were then further characterized using targeted next-generation sequencing to define the genetic profile of locally advanced WDTC.

## Materials and Methods

### Study groups

The study protocol was approved by the University of Pittsburgh Institutional Review Board (PRO12010393), including ethical approval under the University of Pittsburgh Human Research Protections Office. All procedures performed in studies involving human participants were in accordance with the ethical standards of the institutional and/or national research committee and with the 1964 Helsinki declaration and its later amendments or comparable ethical standards. Informed consent was obtained from participants. Under protocol, an institutional review was performed to identify locally invasive thyroid carcinomas (T4) diagnosed in the years 1987–2013, which yielded a total of 80 patients. Cases were restaged according to the 8^th^ edition AJCC. Histopathologic slides were re-reviewed to confirm the diagnosis and cases were excluded if poorly differentiated or anaplastic carcinoma was identified. For well differentiated papillary or follicular carcinoma (WDTC), information on patient age at diagnosis, tumor size, regional lymph node involvement, local/vascular invasion, distant metastasis, histology, treatment course, development of structural recurrence, and survival was collected. A control group of 102 consecutive T1-T2 WDTC treated with surgical excision at our institution was used as a clinical comparison cohort.

### Molecular analysis

In the study cohort, 26 patients had tissue available for targeted next-generation molecular sequencing which was performed utilizing ThyroSeq v2 assay that tests for mutations in 14 thyroid cancer-related genes and 42 types of gene fusions^7^. All 102 cases in the comparison group were analyzed using the same molecular approach.

### Statistical analysis

Statistical analyses were performed using SPSS version 22 (IBM Corp., Armonk, NY), GraphPad InStat 3 (GraphPad Software, La Jolla, CA, USA), and R version 3.0.0 (R Foundation for Statistical Computing, Vienna, Austria). The user-written package “cmprsk” was used to analyze cumulative incidence with the Fine-Gray test. Continuous variables were summarized by median and interquartile range, and they were compared between groups using the Mann-Whitney U test. Chi-squared or Fisher’s exact test were used to determine association between categorical variables. Patient survival time was analyzed by Kaplan-Meier methods and compared between groups via the log-rank test. Time to recurrence, treating death as a competing risk, was analyzed by cumulative incidence and compared between groups via the Fine-Gray test. A p-value <0.05 was used to determine statistical significance.

## Results

**Figure 1:**
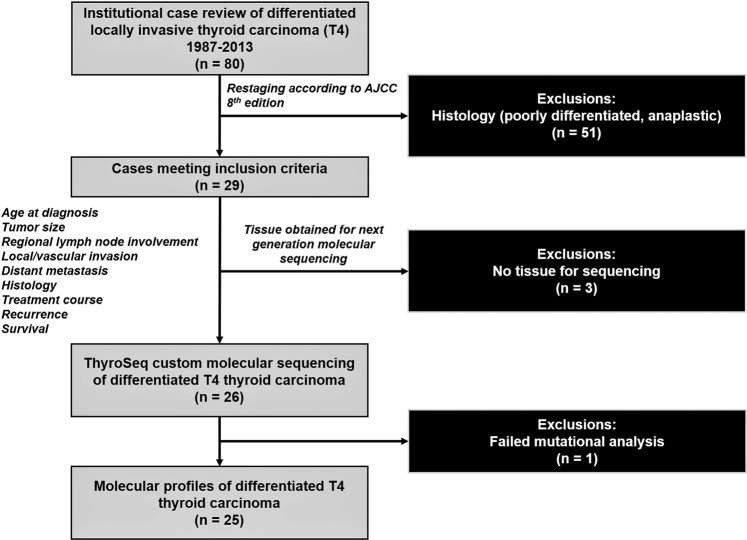
Flow Diagram of Case Selection and Study Cohort. A schematic demonstrating the inclusion and exclusion criteria used to identify the patient cohort.

Among the roughly 180 patients with well-differentiated thyroid cancer seen at our institution per year from 1987 to 2013, 80 patients were managed surgically for well-differentiated T4 thyroid cancer, and 26 (36%) met criteria for inclusion into the study. Of the 26 tissue samples available for sequencing, one failed mutational analysis due to storage-related degradation of the genomic material, resulting in 25 patient samples with genomic sequencing evaluable (Fig. 1). Demographic and clinicopathological characteristics of the study cohort are illustrated in Table 1. There were 16 females and 9 males. Mean age at diagnosis was 58 years and patients were followed for a mean of 60 months (1–191). Histopathologic analysis of these tumors revealed conventional papillary thyroid carcinoma (PTC) in 12 (48%) cases, tall cell variant of papillary thyroid carcinoma (TCV-PTC) in 10 (40%), and Hürthle cell carcinoma (HCC) in 3 (12%). Mean tumor diameter measured 4.4 cm (0.7–11.8 cm), and 20 (95%) patients demonstrated regional lymph node involvement, with an average of 8 (1–26) pathological lymph nodes. Twenty patients were staged as T4a and five patients were staged as T4b. A total of 11 (44%) patients developed recurrent disease. There were 9 (36%) patients with recurrences in the neck and 3 of these patients also developed distant metastasis (two patients with pulmonary metastasis and one patient with widespread liver, brain, and spine metastasis). Two of these 11 patients without local recurrence developed pulmonary metastasis. Overall distant metastases were present in 5 (20%) patients at a range of 5 to 37 months after diagnosis. Of the 5 patients with distant metastasis four harbored a co-mutation with TERT c228T and BRAF. Six of the 25 (24%) patients died of disease.

**Table 1:** Demographic Characteristics of T4 Differentiated Thyroid Carcinoma.

Characteristic	Data
Subjects, n	25
Age, years, mean (range)	58 (29–86)
Sex, male, n (%)	9 (36)
Sex, female, n (%)	16 (64)
Tumor diameter, cm, mean (range)	4.4 (0.7–11.8)
Unknown, n	1
**Regional lymph node involvement**	
Positive, n (%)	20 (95)
Negative, n (%)	1 (5)
Unknown, n	4
Number of regional lymph nodes involved, mean, (range)	8 (1–26)
Local invasion, n (%)	25 (100)
**Distant metastasis (at diagnosis)**	
Present, n (%)	3 (17)
Absent, n (%)	15 (83)
Unknown, n	7
Follow-up, months, mean (range)	60 (1–191)

**Figure 2:**
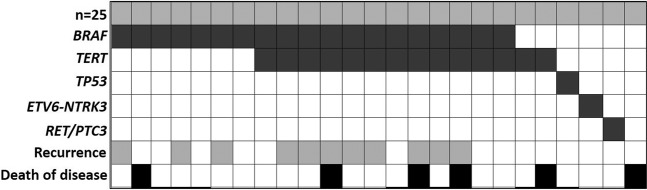
The Mutational Profile of T4 WDTC. Locally aggressive WDTC have a distinct molecular profile. Each patient is depicted as a column, and every parameter is shown in the rows. BRAF and TERT co-mutations occur in 12 (48%) of locally aggressive WDTC.

Molecular analysis revealed alterations in 24/25 (96%) T4 tumors (Fig. 2). The most common mutation was *BRAF* V600E, present in 19 (76%) tumors. The second most frequently identified mutation involved the promoter region of the *TERT* gene and was found in 14 (56%) of T4 tumors. This included C228T *TERT* mutation in 9 tumors and C250T mutation in 5 tumors. Co-occurrence of *TERT* and *BRAF* V600E mutations was identified in 12 (48%) of stage IV WDTC, TERT was the only identified alteration in 2 (8%) of study group tumors. Other alterations included *TP53* mutation, *ETV6-NTRK3* fusion, ELE1/RET fusion, and *RET/PTC3* fusion, all found in single cases.

Within our cohort of T4 tumors, those carrying *BRAF* V600E mutation revealed no significant univariable correlation with patient age, gender, tumor size, regional lymph node or distant metastasis. However, among patients with regional lymph node involvement, those with tumors that harbored *BRAF* V600E had a median of 8 positive lymph nodes compared to a median of 2 positive lymph nodes in tumors negative for this mutation, a difference that bordered statistical significance (p = 0.073). The mean number of lymph nodes examined in BRAF + and BRAF- cases were nearly equivalent at 25.8 and 24.5, respectively.

**Figure 3:**
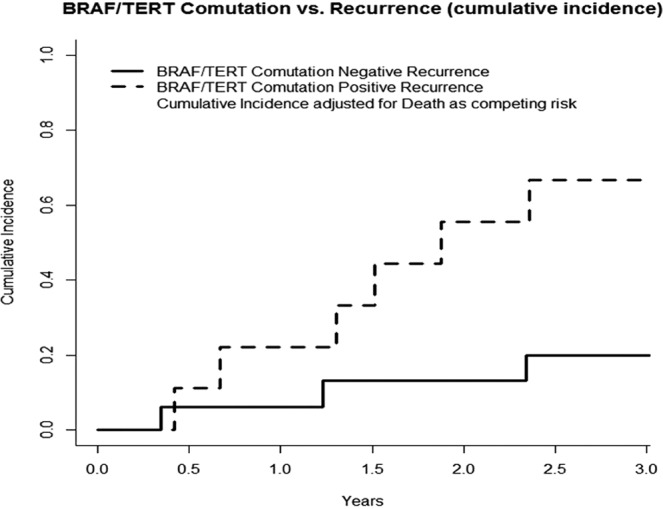
Incidence of Recurrence by Mutation Status. Occurrence of BRAF and TERT co-mutations are associated with disease recurrence. Patients with co-mutations are at significantly higher risk of suffering from recurrence. Cumulative incidence of recurrence was calculated with death as a competing risk.

In T4 tumors carrying both *BRAF* V600E and *TERT* mutations, no significant associations with patient age, gender, or clinicopathological parameters were identified. However, analysis of tumor recurrence by the Fine-Gray Test, adjusted for death as a competing risk, revealed that the presence of these two mutations was associated with a significantly increased risk of recurrence, with a 3-year cumulative incidence of 58.3% versus 18.3% (*p* = *0.04*) (Table 2, Fig. 3). Patients who harbored only the C228T *TERT* mutation experienced particularly high recurrence rates, with a 5-year cumulative incidence of 80% (*p* = *0.02*). Kaplan-Meier survival analysis did not demonstrate significant differences in survival in patients with tumors carrying the *BRAF* V600E mutation or *BRAF* and *TERT* mutations.

**Table 2:** Correlation between specific mutation and recurrence risk in T4 well differentiated thyroid cancer.

	Mutation Positive, estimated 3-year recurrence rate	Mutation Negative, estimated 3-year recurrence rate	p-value
BRAF V600E positive (n = 7)	47.37%	0.00%	p = 0.04*
TERT positive (n = 2)	50.00%	19.70%	p = 0.22
BRAF and TERT positive (n = 12)	58.30%	16.48%	p = 0.05*

We next sought to determine if the mutational profile of T4 cancers was distinct from that of early stage cancers using a series of 102 consecutive T1-T2 cancers treated with surgical excision at our institution. PTC was the predominant diagnosis (91/102, 89%); follicular carcinomas comprised the remainder (11/102, 11%). The results of molecular analysis are shown in Table 3 and reveal, as expected, that mutations in *BRAF* and the *RAS* family of genes were the most commonly identified genetic alteration in early-stage WDTC, overall consistent with the findings of previous studies^6^. *TERT* mutations were found in 8 (8%) of the 102 T1-T2 tumors, and only 5 (5%) contained both *BRAF* V600E and *TERT* mutations. Overall, advanced T4 thyroid cancers more frequently harbored *TERT* mutations and had a 10-fold higher rate of co-occurring *BRAF* and *TERT* mutations (Fig. 2, Table 3).

**Table 3:** Mutations in a consecutive series of 102 T1/T2 differentiated thyroid carcinomas.

Mutation type	Number of cases
BRAF V600E	45
NRAS	17
HRAS	9
KRAS	8
PTEN	1
TERT	8
TERT only	3
TERT + BRAF	5
RET/PTC	4
PAX8/PPARG	1
ETV6/NTRK3	2
Mutation negative	13
**TOTAL cases**	**102**

## Discussion

In this study, we provide genetic characterization of locally advanced T4 well differentiated thyroid cancer and demonstrate that the majority (19/25, 76%) are driven by *BRAF* V600E mutation and 12 (48%) also harbor co-existent *TERT* promoter mutations. Recurrence was particularly high in T4 WDTC with coexistent TERT C228T promoter mutations and thus, this genetic profile may allow further prognostication of WDTC.

Specifically, we found that the incidence of common driver mutations in locally advanced differentiated thyroid cancer is high, with 24 of 25 tumors (96%) harboring at least one mutation. Interestingly, two-thirds of T4 tumors harbored the *BRAF* V600E mutation and none showed *RAS* mutations, which is the second most common genetic event reported in PTC and FTC. *RAS* mutations are known to occur throughout the spectrum of thyroid cancers including encapsulated follicular variant thyroid cancers without nodal disease, as well as in dedifferentiated or anaplastic cancers associated with distant metastasis^8,9^. The results of this study provide evidence that the majority of well differentiated T4 thyroid cancers have mutations in *BRAF* and/or TERT promoter regions.

Another difference in the molecular profile of T4 tumors as compared to the control cohort with T1/T2 tumors was that *TERT* mutations were observed in over half as compared to less than 10% of lower stage thyroid cancers (p < 0.05). The latter is like the incidence previously reported in the general population of WDTC^10,11^. In fact, the incidence of *TERT* mutations we observed in T4 well differentiated thyroid cancers was like that reported in poorly differentiated and anaplastic thyroid carcinomas. For example, Landa *et al*. identified *TERT* mutations in 50% of ATC and PDTC, 22% of PTC, and 25% of HCC^12^. A similar study by Liu *et al*. reported observed frequencies of *TERT* promoter mutations (C228T and C250T) in 50% of ATC, 25% of PTC, 22% of FTC, and 0% MTC^13^. However, our observed incidence of *TERT* promoter mutations is noted to be greater than that observed by Song *et al*., who noted 13% of patients in their cohort of stage III/IV patients possessed *TERT* promoter mutations. Interestingly, the incidence in stage I/II patients was also noted to be lower at 1.7% which perhaps reinforces the known geographic/ethnic variation in mutational landscapes^14^.

The 48% frequency of co-occurrence of *TERT* and *BRAF* mutations in our cohort of T4 appears to be higher than the observed frequency described in papillary, poorly differentiated, and anaplastic thyroid carcinoma^11,12,15^ as well as analyses completed in other geographic populations^14^. The *TERT*/*BRAF* genotype in this cohort of T4 was nearly 7-fold higher than that seen in the lower risk PTC and CPTC cohort described by Xing *et al*^15^. These studies all provided initial evidence that TERT mutations may be associated with aggressive disease. Our findings provide further support to the notion that the development of aggressive thyroid carcinoma is driven by the acquisition of multiple mutations which cooperate to enhance tumor growth and spread.

In this cohort of T4 cases, the presence of *BRAF* mutations alone and *BRAF* and *TERT* mutations were associated with an increased risk of disease recurrence. This is consistent with findings in other series of well differentiated thyroid cancer, where *BRAF* mutations in tumors>1 cm in size and particularly co-occurring *BRAF* and *TERT* mutations were associated with a significantly higher risk of disease recurrence^16,17^. A subset of BRAF + thyroid cancers will present with aggressive T4 tumors and or recur, but we do not know why. In our study groups *BRAF* V600E, was present in 19/25 (76%) of T4 tumors and 45/102 (44%) of T1/T2 WDTC tumors. Co-occurrence of TERT/BRAFV600E mutations were present in 12/25 (48%) of T4 and 5/102 (5%) of T1/T2 thyroid carcinomas. There is a building body of evidence that co-mutations of BRAF/TERT signify a highly aggressive form of thyroid cancer that is more likely to recur. We did not find a significant correlation between the presence of these mutations and mortality in this cohort though it has been noted in other studies^14^. This may be due to the relatively small number of deaths observed in this patient cohort. Further, though others^14^ have shown synergy between *TERT* promoter mutations and *RAS* mutations, the lack of *RAS* mutations in our cohort precludes any such interpretation of the data.

Out of the four cases in this cohort that did not have *BRAF* or *TERT* mutations, there were tumors with a *TP53* mutation, *ETV6-NTRK3* fusion, ELE1/RET fusion, and *RET/PTC3* fusion. *TP53* mutations are known to occur more frequently in de-differentiated thyroid cancer, but they have also been found in well differentiated cancers with a tendency to be more aggressive^18^.

This study has a few limitations including the small sample size and retrospective nature. Fortunately, locally advanced WDTC is rare, but this provides few opportunities to study this cohort prospectively. Another limitation is the study group of T4 tumors had a much higher incidence of TCV-PTC, a more aggressive histologic variant, than the control group. Also, there was a low number of WDTC-specific deaths limiting the survival analysis. A prospective multicenter study on the molecular phenotype and survival of advanced WDTC is needed. However, it must be noted that studies of over 388 patients with WDTC in the TCGA by Shen *et al*. supported a model that predicted poor clinical outcomes based on mutational status^19^. There they posit that possessing *BRAF* V600E or *RAS* mutations in conjunction with *TERT* promoter mutations portend higher risk than *BRAF* V600E or *TERT* promoter mutations alone and that these mutations are poorer prognosticators as compared to *RAS* mutations alone.

In summary, the results of this study show that T4 well differentiated thyroid cancer develops primarily via *BRAF* V600E-initiated pathway, and more than half of them also carry *TERT* mutations. Furthermore, we show that co-occurrence of *BRAF* and *TERT* may serve as a marker for highly aggressive locally invasive thyroid carcinoma with an increased risk for recurrence particularly if associated with C228T and, if found preoperatively, such genetic profile could influence patient management. Further work is needed to determine if the co-occurrence of these mutations is also associated with a risk of recurrence and mortality in T4 thyroid cancer in a larger cohort of patients. In addition, characterization of recurrent/metastatic lesions to identify possible transformation and/or differences in molecular profile in these well-differentiated thyroid cancers is needed.
